# An isoflavone-enriched diet alleviates Parkinson’s disease in mice by inhibiting ferroptosis through gut microbiota-mediated serotonin production

**DOI:** 10.3389/fimmu.2026.1730833

**Published:** 2026-02-03

**Authors:** Le Yang, Yan Ma, Haiying Wang, Likai Xie, Yan Yu

**Affiliations:** 1 School of Public Health, Xi’an Jiaotong University Health Science Center, Xi’an, Shaanxi, China; 2 Jilin Provincial People’s Hospital, Changchun, Jilin, China

**Keywords:** 5-HT, ferroptosis, isoflavones, *L. intestinalis*, Parkinson’s disease

## Abstract

**Objective:**

Parkinson’s disease (PD) is a severe neurodegenerative disorder whose pathogenesis is closely linked to gut microbiota dysregulation. However, whether and how modulation of gut homeostasis can ameliorate PD remains unclear. Dietary isoflavones have been associated with neuroprotective effects and show strong potential in shaping the composition of the gut microbiota, yet their underlying mechanisms in PD are poorly understood.

**Methods:**

Microbiomics and non-targeted metabolomics were utilized to characterize microbial community composition and metabolic alterations in the MPTP (1-methyl-4-phenyl-1,2,3,6-tetrahydropyridine)-induced mouse model of PD. Subsequently, both the MPTP-induced PD mouse model and *in vitro* cell experiments were employed to investigate the effects and underlying mechanisms of the differentially regulated metabolite serotonin on PD pathogenesis.

**Results:**

Here, we show that an isoflavone-enriched diet alleviates motor deficits and dopaminergic neuron loss in an MPTP-induced mouse model of PD. This protective effect is mediated via a gut–brain axis mechanism: isoflavones promote the expansion of intestinal *Lactobacillus* species, especially *Lactobacillus intestinalis*, leading to increased 5-hydroxytryptamine (5-HT) production in both serum and brain. Elevated 5-HT activates central 5-HT1A receptor (5-HTR1A), which in turn triggers downstream PI3K-AKT signaling to suppress ferroptosis—a key pathogenic process in PD. Pharmacological inhibition of either 5-HTR1A or the PI3K-AKT pathway abolishes the neuroprotective effects of 5-HT.

**Conclusions:**

Our findings reveal a novel dietary-microbiota-serotonergic pathway that mitigates ferroptosis and neurodegeneration, highlighting the therapeutic potential of isoflavone-based interventions for PD.

## 1 Introduction

Parkinson’s disease (PD) is the fastest-growing neurodegenerative disorder worldwide, with prevalence increasing sharply with age (1). It is characterized by the loss of dopaminergic neurons in the substantia nigra (SN), resulting in core motor symptoms—bradykinesia, tremor, and rigidity—and the accumulation of misfolded α-synuclein into Lewy bodies (1, 2). The current pharmacological cornerstone, levodopa, primarily alleviates dopaminergic symptoms but does not halt underlying neurodegeneration, highlighting an urgent need for disease-modifying therapies that target novel pathological pathways. In recent years, the gut-brain axis has emerged as a key contributor to PD pathogenesis, opening new avenues for therapeutic intervention.

Compelling evidence underscores the role of the gut microbiota in modulating brain health and disease (3, 4). Alterations in the gut microbiota have been consistently observed in patients with PD (5). For instance, abundance of Prevotellaceae is significantly reduced, potentially compromising mucin production and gut barrier integrity, thereby promoting inflammation and α-synuclein misfolding (5, 6). Conversely, increased levels of Enterobacteriaceae are associated with greater motor symptom severity (7). Transplantation of gut microbiota from PD patients into α-synuclein-overexpressing mice exacerbates motor deficits and neuroinflammation compared to microbiota from healthy controls (6). Furthermore, microbial metabolites such as short-chain fatty acids (SCFAs) may modulate neuroinflammation; reduced fecal SCFA levels (e.g., butyrate) have been reported in PD patients, which could impair regulatory T-cell function and promote neuroinflammatory processes (8). Additionally, supplementation with *Bifidobacterium animalis* subsp. lactis Probio-M8 has been shown to alleviate PD symptoms by increasing serum acetate and dopamine levels (9). Collectively, these findings confirm the involvement of the gut microbiota in PD pathogenesis. However, whether and how modulation of the gut microbiota can attenuate PD progression remains unclear.

Dietary components are among the primary factors regulating the gut microbiota. Among various dietary constituents, isoflavones—phytoestrogens abundant in soybeans and other legumes—have been shown to modulate the gut microbiota and have garnered significant interest due to their potential neuroprotective properties (10–12). Epidemiological observations have long noted a lower prevalence of PD in Asian populations, where traditional diets are rich in isoflavones, suggesting a possible protective effect (13, 14). Isoflavones such as genistein and daidzein exhibit antioxidant, anti-inflammatory, and estrogen receptor-modulating activities (15, 16). However, their bioavailability and biological activity are highly dependent on metabolism by the gut microbiota into more potent derivatives, such as equol (17). This metabolic capacity is not universal and is mediated by specific gut bacteria, particularly certain species within the *Lactobacillus* genus (17, 18). In addition to being converted into bioactive metabolites, isoflavones also shape the gut microbial composition, thereby promoting the production of other neuroprotective substances. For example, isoflavone-enriched *Lactobacillus* can metabolize tryptophan and, in collaboration with host cells, enhance serotonin (5-hydroxytryptamine, 5-HT) synthesis (19, 20). Patients with PD exhibit reduced 5-HT levels in brain tissue, and increased 5-HT may regulate dopamine, γ-aminobutyric acid (GABA), and glutamate through its receptors, including the 5-HT1A receptor, potentially improving both motor and non-motor symptoms of PD (21–23).

Ferroptosis, an iron-dependent form of regulated cell death driven by uncontrolled lipid peroxidation, has emerged as a critical mechanism underlying dopaminergic neuron loss in PD (24). A key defense against this process is glutathione peroxidase 4 (GPX4), which is highly expressed in neurons and functions as the primary enzyme that reduces cytotoxic lipid hydroperoxides to non-toxic lipid alcohols, using glutathione as a cofactor (24, 25). Conditional knockout of GPX4 in mouse neurons leads to rapid ferroptosis and neurodegeneration, recapitulating key features of PD pathology (26). Importantly, the SN of PD patients frequently exhibit reduced levels of GPX4 (27). The PI3K-AKT signaling pathway, a well-established master regulator of cell survival, has recently been identified as a crucial upstream modulator of GPX4 and ferroptosis (28). For example, activation of AKT has been shown to phosphorylate and activate the transcription factor Nrf2—a master regulator of the antioxidant response—in the MPTP mouse model of PD (29). This results in the transcriptional upregulation of not only GPX4 but also genes involved in glutathione synthesis, such as xCT (the catalytic subunit of system Xc−), thereby establishing a robust cellular defense network against lipid peroxidation (29). Tryptophan metabolism-derived 5-HT has been implicated in the inhibition of ferroptosis (30). However, whether 5-HT alleviates PD by regulating ferroptosis and the underlying mechanisms remain unclear.

In this study, we demonstrate that an isoflavone-enriched diet alleviates motor deficits and dopaminergic neuron loss in MPTP-induced PD in mice through modulation of the gut microbiota. Specifically, the isoflavone-enriched diet increases fecal abundance of *Lactobacillus*, leading to elevated levels of 5-HT in both serum and brain tissue. Administration of *Lactobacillus intestinalis* (*L. intestinalis*) or 5-HT precursor, 5-hydroxytryptophan (5-HTP), similarly increases 5-HT levels and alleviates PD symptoms in mice. Mechanistically, 5-HT activates neuronal 5-HT1A receptor (5-HTR1A), resulting in upregulation of the PI3K-AKT signaling pathway, enhanced expression of GPX4, and suppression of neuronal ferroptosis. Our findings reveal a gut–microbiota–brain axis that mediates neuroprotection, positioning dietary isoflavone intervention as a promising strategy for the prevention and treatment of PD.

## 2 Materials and methods

### 2.1 Animal and treatments

Male C57BL/6 mice (8 weeks old) were maintained under a 12-hour light/dark cycle with free access to feed and water. To induce PD, mice were intraperitoneally injected with 1-methyl-4-phenyl-1,2,3,6-tetrahydropyridine (MPTP, 15 mg/kg; Sigma-Aldrich) for five consecutive days (31, 32). For isoflavone treatment, mice were fed either an isoflavone-enriched diet [genistein (0.24 g/kg diet) and daidzein (0.22 g/kg diet)] or an isoflavone-free diet (Envigo, Indianapolis, IN) ad libitum for four weeks (10, 12). To deplete gut microbiota during isoflavone treatment, mice received sterile water supplemented with vancomycin (0.5 g/L), neomycin (1 g/L), metronidazole (1 g/L), and ampicillin (1 g/L) for four weeks, concurrently with the isoflavone or isoflavone-free diet. In the fecal microbiota transplantation (FMT) experiment, recipient mice were orally administered antibiotics (200 mg/kg ampicillin, neomycin, and metronidazole; 100 mg/kg vancomycin) for five consecutive days to eliminate commensal microbiota (33, 34), followed by a one-day washout period with normal water. Fecal samples from six donor mice were pooled and used for FMT, which was performed three times per week for three weeks as previously described (33, 34). For *L. intestinalis* intervention, mice were first depleted of commensal microbes via oral administration of antibiotics (200 mg/kg ampicillin, neomycin, and metronidazole; 100 mg/kg vancomycin) for five consecutive days (33, 34), then pretreated with *L. intestinalis* (2 × 10^8^ CFU/mouse) daily for four weeks prior to MPTP challenge (12). For 5-HTP treatment, mice received 5-HTP at a dose of 1 g/kg/day in food pellets for four weeks (35). Briefly, this corresponded to 6.7 mg 5-HTP per gram of chow, prepared by mixing finely crushed chow with 5-HTP and a cellulose binder, followed by drying into pellets (35). For 5-HTR1A inhibition, mice were subcutaneously injected with WAY-100635 (1 mg/kg) daily during 5-HTP treatment (36, 37).

### 2.2 Cell culture and treatments

SH-SY5Y cells were purchased from ATCC (#CRL-2266, American Type Culture Collection) and cultured in Dulbecco’s Modified Eagle’s Medium/Nutrient Mixture F-12 (DMEM/F12, Hyclone), supplemented with 10% fetal bovine serum (FBS; BI) and antibiotics (100 U/mL penicillin, 100 μg/mL streptomycin; Thermo Fisher Scientific, 15140122) at 37°C in a humidified atmosphere containing 5% CO_2_. All experiments were performed at low passage numbers (between passage 5 and 15). For each experiment, cells were seeded in 6-well plates and incubated for 24 h, followed by removal of antibiotics. Cells were then pretreated with 10 μM 5-HT for 2 h prior to exposure to MPP^+^ (1 mM; Sigma, D048) (30). In ferroptosis inhibition experiments, cells were cotreated with 10 μM 5-HT and Fer-1 (10 μM) for 2 h before MPP^+^ challenge (16). For PI3K inhibition, cells were pretreated with 10 μM 5-HT and PKI-402 (1 μM) for 2 h prior to MPP^+^ treatment (28). For AKT inhibition, cells were pretreated with 10 μM 5-HT and A-443654 (10 μM) for 2 h before MPP^+^ exposure (38). To inhibit 5-HTR1A, cells were pretreated with WAY-100635 (1 μM) in combination with 10 μM 5-HT for 2 h prior to MPP^+^ treatment (36, 39). Subsequently, cells were exposed to MPP^+^ (1 mM) for 24 h and then harvested for further analysis.

### 2.3 Bacteria culture

*L. intestinalis* strains (ATCC 49335) were obtained from the American Type Culture Collection and cultured in MRS broth (Hopebio, China) at 37 °C under anaerobic conditions. Bacterial cell pellets were harvested by centrifugation, washed, and resuspended in oxygen-free phosphate-buffered saline (PBS) to a final concentration of 1 × 10^9^ CFU/mL.

### 2.4 Cell viability assay

Cell viability was assessed using the Cell Counting Kit-8 (CCK-8) assay. Briefly, cells were seeded at a density of 1 × 10^4^ cells per well in 96-well plates and cultured at 37°C for 24 h. Cells were then treated with 1 mM MPP^+^ (Sigma, D048) alone or in combination with 10 μM 5-HT, PKI-402 (1 μM), A-443654 (10 μM), WAY-100635 (1 μM), or 10 μM Fer-1 for 24 h as described above. Subsequently, culture medium was replaced with CCK-8 solution, and cells were incubated for 2 h according to the manufacturer’s instructions (Solarbio, China). The absorbance was measured at 490 nm using a microplate reader.

### 2.5 Behavioral tests

To evaluate the effects of different treatments on behavioral deficits in MPTP-induced PD in mice, animals were assessed using the beam traversal, pole test, rotarod test, hindlimb clasping test, and gait analysis as previously described (32, 40).

For the beam traversal test, a 1-m-long beam was divided into four segments, each 0.25 m in length, with decreasing widths of 3.5 cm, 2.5 cm, 1.5 cm, and 0.5 cm. Each segment had 1-cm overhangs positioned 1 cm below the beam surface. The widest segment served as a loading platform, while the narrowest end was connected to the home cage. Mice underwent two days of training to traverse the beam before testing. On day 1, mice performed one trial with the home cage placed close to the loading platform and were gently guided forward along the narrowing beam. They then completed two additional trials with minimal or no assistance to promote independent movement and balance. On day 2, mice completed three trials without assistance. On day 3, performance was tested over three trials, during which the time to traverse from the loading platform to the home cage was recorded. Timing began when the mouse placed its forelimbs onto the 2.5-cm segment and ended when one forelimb reached the home cage.

The pole test was conducted as previously described (40). Briefly, after acclimatization in the behavioral testing room for at least 30 min, mice were placed near the top of a vertical pole (75 cm long, 9 mm in diameter), positioned 7.5 cm from the top, and oriented head-upward. The following parameters were recorded: time to turn downward, time to climb down, and total descent time (in seconds).

For the rotarod test, mice were placed on an accelerating rotating rod, and the duration they remained on the device was measured. Over a 5-minute period, the rotation speed gradually increased from 4 to 40 rpm. A trial ended if the mouse fell off or grasped the rod and spun around twice without attempting to walk. Motor performance was expressed as the percentage of the average latency to fall across three trials relative to the control group.

For the hindlimb clasping test, mice were gently lifted by the midsection of the tail and observed for 5–10 s. Hindlimb posture was scored as follows: 0 (no clasping; limbs freely moved and extended outward), 1 (one hindlimb clasped inward or both limbs showed partial inward retraction), 2 (both limbs consistently clasped inward for most of the observation period but retained some flexibility), or 3 (immediate and sustained clasping with no limb extension or movement).

### 2.6 Lipid ROS detection

Lipid ROS assay was performed as previously (32, 41). Briefly, cells were treated with MPP^+^ and inhibitors as mentioned above. After treatment, cells were incubated with C11-BODIPY 581/591 (10 μM) for 30 min at 37°C. Following three washes with PBS, lipid ROS levels were assessed using a confocal laser scanning microscope (Olympus, Tokyo, Japan) with excitation wavelengths of 488 nm and emission detected at 594 nm. The lipid ROS-positive area was quantified using ImageJ software (version 1.51).

### 2.7 GSH, iron and MDA assays

GSH, Fe^2+^ and MDA levels were measured by GSH assay kit (A006-2, Nanjing Jiancheng Bioengineering Institute, China), Iron Assay Kit and MDA Assay kit (MAK085, Sigma-Aldrich, USA) according to the manufacturer’s instructions. Briefly, for GSH detection, tissues or incubated cells were collected and homogenized in PBS, then centrifuged at 3000 × g for 10 min at 4°C, and the supernatants were collected for measurement at 420 nm. For Fe²^+^ determination, samples were homogenized in the provided assay buffer and centrifuged at 16,000 × g for 10 min at 4°C. Clarified supernatants (50 μL) were transferred to a 96-well plate, followed by the addition of 5 μL of assay buffer. After a 30-min incubation in the dark at 25°C, 100 μL of iron probe was added to each well, and the incubation continued for an additional 60 min under the same conditions. Absorbance was measured at 593 nm, and concentrations were calculated based on a standard curve. For MDA detection, samples were homogenized on ice in 300 μL of MDA Lysis Buffer supplemented with 3 μL of BHT (100×), then centrifuged at 13,000 × g for 10 min, and the supernatants were used for analysis.

### 2.8 Detection of 5-HT concentration

5-HT levels were determined as previously described (42). Briefly, chromatographic analysis was performed using a Thermo Fisher Accucore Polar Premium column (2.6 μm, 2.1 mm × 100 mm) maintained at a column temperature of 35°C. Separation was achieved with a linear gradient elution at a flow rate of 0.2 mL/min. The mobile phase consisted of 0.1% acetic acid in water (solvent A) and acetonitrile (solvent B). The gradient program was as follows: 0–2 min, 10% B; 2–3 min, 10–50% B; 3–4 min, 50–70% B; 4–5 min, 70–95% B; 5–6 min, 95% B; 6–7 min, 95–70% B; 7–8 min, 70–50% B; 8–9 min, 50–10% B; 9–10 min, 10% B (stop). The autosampler was maintained at 4°C. Mass spectrometric detection was carried out in parallel reaction monitoring (PRM) mode. The following precursor-to-product ion transitions were monitored for 5-HT: m/z 177.10210 [M+H]+ → 160.07561, with a retention time of 1.08 min (qualifier ion at m/z 160.07561, RT: 1.01 min).

### 2.9 RNA extraction and qPCR

Tissue RNA extraction and qPCR were performed as previously described (43, 44). Briefly, tissues from the STR were homogenized in TRIzol reagent (Invitrogen, Carlsbad, CA, USA), followed by phase separation with chloroform, RNA precipitation with isopropanol, and washing with 75% ethanol. RNA concentration and purity were determined using a spectrophotometer. cDNA was synthesized using the cDNA Synthesis SuperMix (AE311-02, TransGen Biotech, China) according to the manufacturer’s instructions. qPCR was carried out using FastStart Universal SYBR Green Master Mix (ROX) (Roche, Basel, Switzerland) on a StepOnePlus Real-Time PCR System (Applied Biosystems, Foster City, CA, USA) under conditions as previously reported (41). The primers used in this study were as follows: PTGS2 (forward: 5′-TGAGCAACTATTCCAAACCAGC-3′; reverse: 5′-GCACGTAGTCTTCGATCACTATC-3′) and GAPDH (forward: 5′-AACTTTGGCATTGTGGAAGG-3′; reverse: 5′-ACATCATCCCTGCATCCACT-3′). Gene expression levels were normalized to GAPDH using the 2^−ΔΔCt^ method.

### 2.10 Bacterial DNA extraction and 16S rRNA sequencing

Bacterial DNA extraction and 16S rRNA gene sequencing were performed as previously described (43). Briefly, genomic DNA from microbial communities was extracted from mouse fecal samples using the FastDNA^®^ Spin Kit for Soil (MP Biomedicals, USA). The quality of the DNA extract was verified by electrophoresis on a 1% agarose gel, and DNA concentration and purity were determined using a NanoDrop 2000 UV-vis spectrophotometer (Thermo Scientific, Wilmington, USA). The bacterial 16S rRNA gene hypervariable regions V3–V4 were amplified using primer pairs 338F (5′-ACTCCTACGGGAGGCAGCAG-3′) and 806R (5′-GGACTACHVGGGTWTCTAAT-3′) on an ABI GeneAmp^®^ 9700 PCR thermocycler (ABI, CA, USA). Following PCR amplification, the products were excised from a 2% agarose gel and purified using the AxyPrep DNA Gel Extraction Kit (Axygen Biosciences, Union City, CA, USA), followed by quantification with a Quantus™ Fluorometer (Promega, USA). Purified amplicons were pooled in equimolar ratios and subjected to paired-end sequencing on an Illumina MiSeq PE300 or NovaSeq PE250 platform (Illumina, San Diego, USA). Operational taxonomic units (OTUs) were clustered at a 97% similarity threshold using UPARSE (version 7.1), and chimeric sequences were identified and removed. Taxonomic classification of OTU representative sequences was performed using the RDP Classifier (version 2.2) against the 16S rRNA database. Principal coordinate analysis (PCoA) based on ANOSIM was used to assess microbial community structure, and linear discriminant analysis effect size (LEfSe) was applied to identify bacterial taxa differentially enriched across groups.

### 2.11 Immunohistochemistry

Tissues for immunohistochemistry were fixed in 4% paraformaldehyde, embedded in paraffin, and sectioned into 5-μm-thick slices. After dewaxing in xylene and rehydration through a graded alcohol series, antigen retrieval was performed using sodium citrate buffer. The sections were then incubated with tyrosine hydroxylase antibody (#AF6113, Affinity, USA; 1:200) according to the manufacturer’s instructions, using the SAP (Mouse/Rabbit) IHC Kit (MXB, China), as previously described (33). TH expression in the STR and SN was visualized under a light microscope (Olympus, Tokyo, Japan). The TH-positive area within the defined signal intensity threshold was quantified using ImageJ software (version 1.51) (31, 40).

### 2.12 Western blotting

Total proteins were extracted using a tissue protein extraction reagent (Thermo Fisher Scientific, USA), and target proteins were separated by SDS-PAGE. Proteins were then transferred onto PVDF membranes and blocked with 5% skim milk in TBST at room temperature for 1 h. The membranes were incubated overnight at 4 °C with primary antibodies against TH (#AF6113, Affinity, USA; 1:1000), 5-HTR1A (AF0482, Affinity; 1:1000), p-AKT (#AF0016, Affinity, USA; 1:1000), PI3K (#AF6241, Affinity, USA; 1:1000), PTGS2 (#AF7003, Affinity, USA; 1:1000), GPX4 (#DF6701, Affinity, USA; 1:1000), and β-actin (#AF7018, Affinity, USA; 1:1000). After washing with TBST, the membranes were incubated with horseradish peroxidase-conjugated Goat anti-Rabbit IgG or Rabbit anti-Mouse IgG secondary antibodies (1:20,000) for 1 h at room temperature. Protein signals were detected using the ECL Plus Western Blotting Detection System (Tanon, China).

### 2.13 Statistical analysis

GraphPad Prism 8.0 was used for statistical analysis. Data are presented as mean ± standard deviation (SD). For comparisons between two groups, a two-tailed unpaired Student’s t-test was applied. For comparisons among more than two groups, one-way analysis of variance (ANOVA) followed by Tukey’s *post hoc* test was performed. Statistical significance was set at *p < 0.05.

## 3 Results

### 3.1 An isoflavone-enriched diet attenuates the MPTP-induced PD-model in mice in a gut microbiota-dependent manner

**Figure 1:**
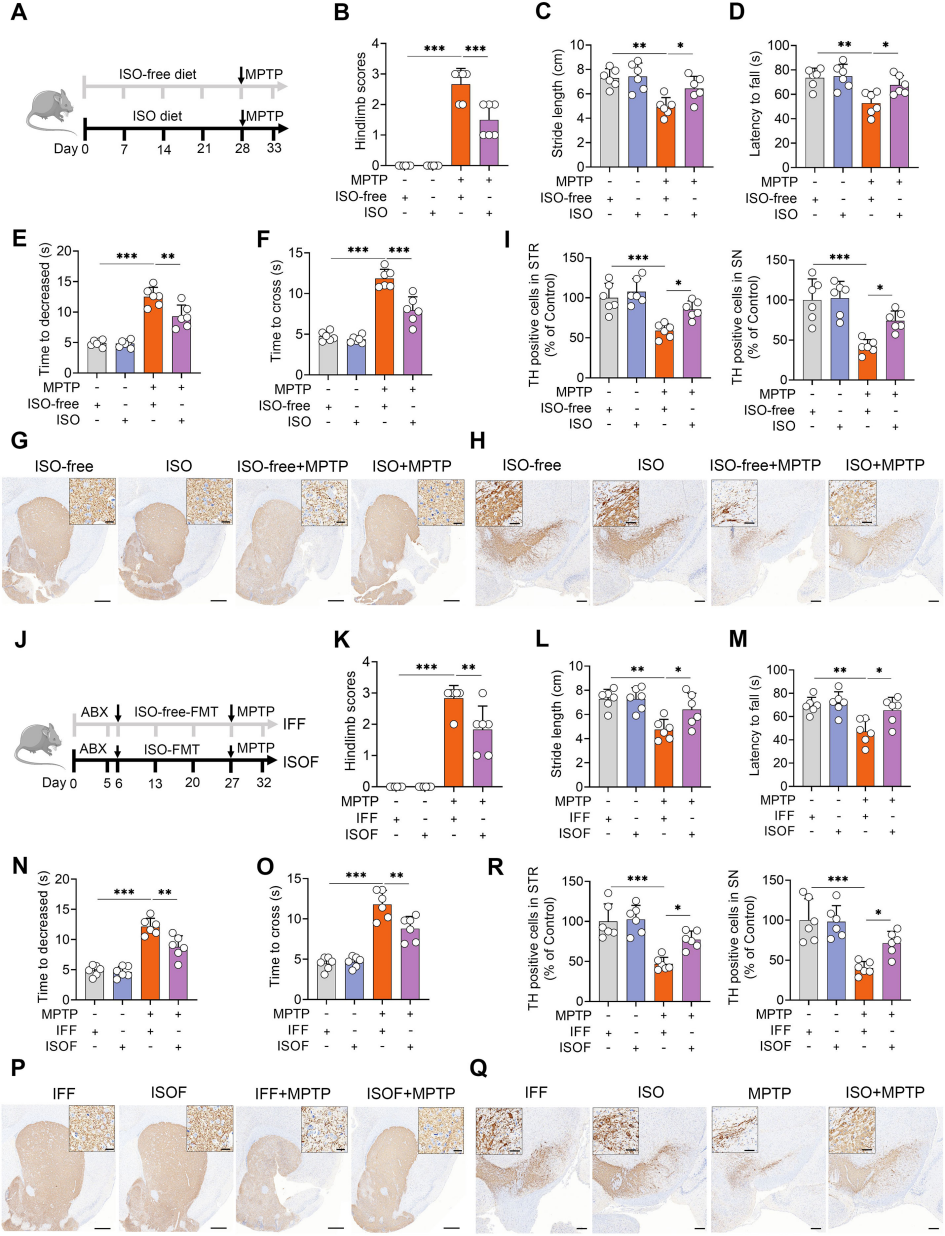
Isoflavone diet alleviates MPTP-induced PD in mice. **(A)** Schematic diagram of isoflavone (ISO) diet treatment. Mice were fed an ISO diet containing genistein (0.24 g/kg of diet) and daidzein (0.22 g/kg of diet) or an ISO-free diet for four weeks, followed by intraperitoneal injection of MPTP (15 mg/kg) for five consecutive days to induce PD (n = 6). **(B–F)** Behavioral assessments including hindlimb clasping scores **(B)**, stride length **(C)**, latency to fall **(D)**, time to descend **(E)**, and time to cross the beam **(F)**. **(G, H)** Representative tyrosine hydroxylase (TH)-stained images in the striatum (STR, **G**) and substantia nigra (SN, **H**). Scale bars: 500 μm for STR (inset, 20 μm) and 200 μm for SN (inset, 50 μm). **(I)** Quantification of TH-positive cells in the STR and SN. **(J)** Schematic diagram of fecal microbiota transplantation (FMT). Mice received oral administration of antibiotics (200 mg/kg ampicillin, neomycin, and metronidazole; 100 mg/kg vancomycin) for five consecutive days to deplete commensal gut microbiota, then switched to water for one day. Fecal samples from six donor mice were pooled and used as inoculum for FMT, which was performed daily for three weeks. This was followed by MPTP (15 mg/kg) injection for five consecutive days to induce PD (n = 6). **(K–O)** Behavioral assessments including hindlimb clasping scores **(K)**, stride length **(L)**, latency to fall **(M)**, time to descend **(N)**, and time to cross the beam **(O)**. **(P, Q)** Representative TH-immunostained images in the STR **(P)** and SN **(Q)**. Scale bars: 500 μm for STR (inset, 20 μm) and 200 μm for SN (inset, 50 μm). **(R)** Quantification of TH-positive cells in the STR and SN. Data are expressed as mean ± SD (n = 6). *p < 0.05, **p < 0.01, and ***p < 0.001 by one-way ANOVA followed by Tukey’s *post hoc* test **(B–F, I, K–O, R)**. IFF, ISO-free diet with FMT; ISOF, ISO diet with FMT.

To investigate the role of isoflavones in MPTP-induced PD, mice were assigned to either an isoflavone-enriched diet (ISO) or an isoflavone-free diet (ISO-free) for four weeks prior to MPTP treatment (10, 12) (**Figure 1A**). The results showed that the abnormal reflex associated with nigrostriatal damage caused by MPTP was attenuated in ISO-treated mice, as indicated by significantly lower hindlimb extension scores in the ISO + MPTP group compared to the ISO-free + MPTP group (**Figure 1B**). Furthermore, ISO administration effectively alleviated MPTP-induced parkinsonian gait disturbances, evidenced by a notable increase in stride length in ISO-treated mice (**Figure 1C**). Motor coordination, balance, and overall neuromuscular function were also improved in ISO-treated mice relative to the ISO-free group following MPTP treatment, as demonstrated by a significantly prolonged latency to fall on the accelerating rotarod test (**Figure 1D**). Additionally, general bradykinesia and motor slowing were reduced: ISO-treated mice exhibited significantly shorter times to traverse a narrow beam (**Figure 1E**) and to descend from a pole (**Figure 1F**), indicating enhanced agility and movement initiation. These findings demonstrate that ISO treatment robustly mitigates a broad spectrum of motor deficits in MPTP-induced PD model in mice. Consistently, immunohistochemical staining revealed that MPTP significantly reduced tyrosine hydroxylase-positive (TH^+^) fibers in the striatum (STR) and TH^+^ dopaminergic neurons in the SN, effects that were reversed in mice receiving the ISO diet (**Figures 1G–I**).

Since the gut microbiota has been closely associated with the development of PD and ISO can modulate the gut microbiota (6, 9, 11, 12), we investigated whether the protective effects of ISO against MPTP-induced PD are mediated through the gut microbiota. We first depleted the gut microbiota using antibiotics (ABX) during ISO intervention (12) (**Supplementary Figure S1A**), and found that ABX-mediated depletion of the gut microbiota attenuated the protective effects of ISO on MPTP-induced motor deficits, as demonstrated by behavioral tests (**Supplementary Figures S1B–F**). Similarly, the ISO-induced increase in TH^+^ cells in both the STR and SN was abolished by ABX treatment (**Supplementary Figures S1G–I**). Next, we performed fecal microbiota transplantation (FMT) from ISO-treated and ISO-free donor mice to recipient mice (**Figure 1J**), and found that recipients receiving FMT from ISO-treated donors (ISOF) exhibited improved motor function compared to those receiving FMT from ISO-free donors (IFF) following MPTP treatment (**Figures 1K–O**). Moreover, ISOF recipients showed higher levels of TH^+^ cells in the STR and SN than IFF recipients in the context of MPTP-induced PD (**Figures 1P–R**). Collectively, these results demonstrate that an isoflavone-enriched diet alleviates the MPTP-induced PD-model in a gut microbiota-dependent manner in mice.

### 3.2 ISO alleviated the MPTP-induced PD-model by increasing *L. intestinalis* in mice

**Figure 2:**
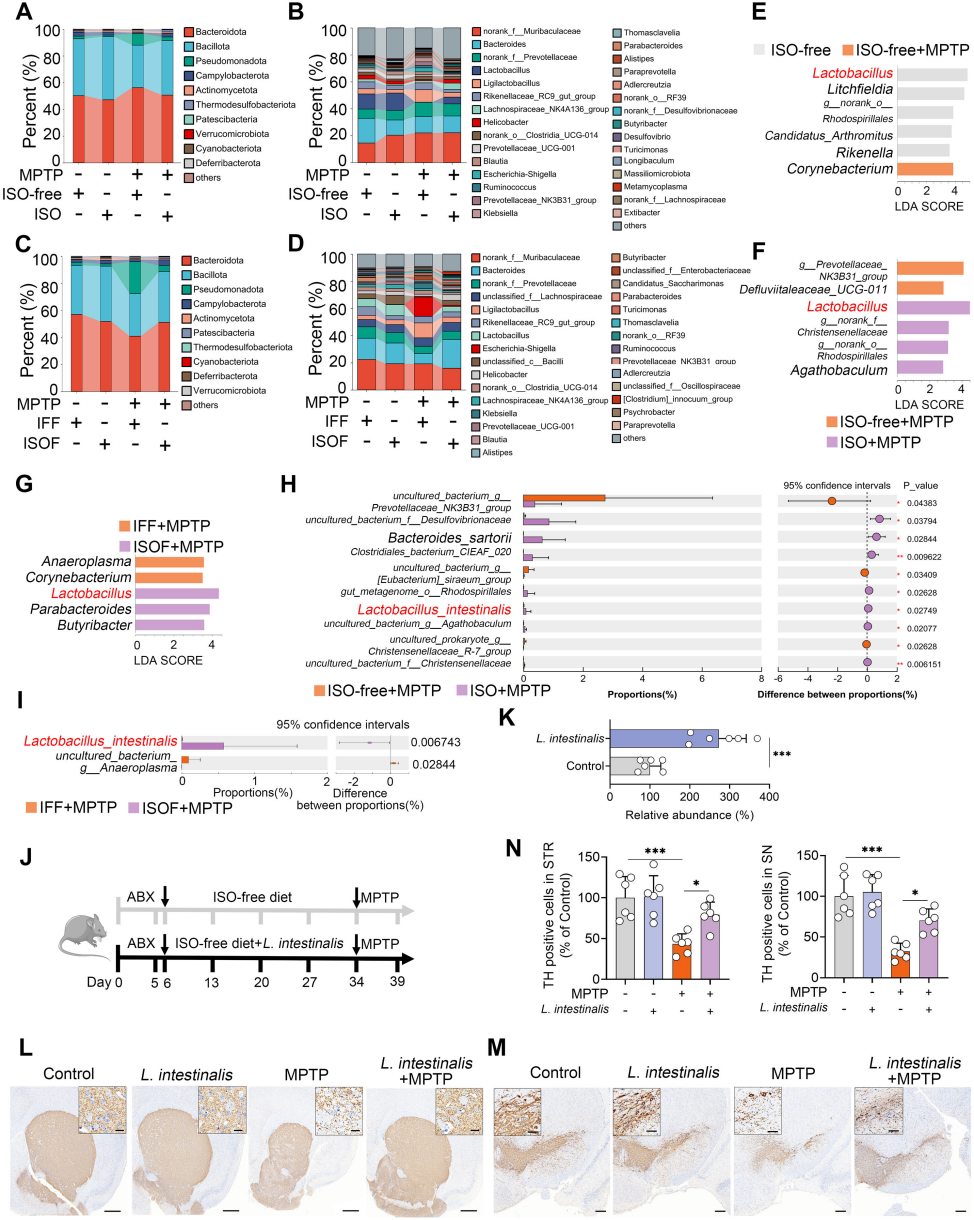
Isoflavone diet-facilitated *Lactobacillus intestinalis* ameliorates MPTP-induced PD in mice. **(A, B)** Gut microbial composition at the phylum **(A)** and genus **(B)** levels in ISO-treated mice. **(C, D)** Gut microbial composition at the phylum **(C)** and genus **(D)** levels in mice receiving different FMT. **(E–G)** LEfSe analysis identifying differential gut microbial taxa between groups. **(H, I)** Student’s t-test identified potential bacterial species differing between ISO-treated and ISOF-treated mice. **(J)** Schematic diagram of *L. intestinalis* treatment. All mice were administered antibiotics (200 mg/kg ampicillin, neomycin, and metronidazole; 100 mg/kg vancomycin) orally for five consecutive days to deplete commensal gut microbiota, followed by pretreatment with *L. intestinalis* (2 × 10^8^ CFU/mouse) for four weeks prior to MPTP administration (n = 6). **(K)** Fecal abundance of *L. intestinalis* was quantified using qPCR in different groups. **(L, M)** Representative TH-stained images in the STR **(L)** and SN **(M)** from *L. intestinalis*-treated mice. Scale bars: 500 μm for STR (inset, 20 μm) and 200 μm for SN (inset, 50 μm). **(N)** Quantification of TH-positive cells in the STR and SN. Data are expressed as mean ± SD (n = 6). *p < 0.05, and ***p < 0.001 by two-tailed unpaired Student’s t-test **(H, I, K)** and one-way ANOVA followed by Tukey’s *post hoc* test **(N)**.

We next investigated which gut microbiota mediates the protective effects of ISO against MPTP-induced PD. ISO treatment had minimal impact on gut microbial alpha diversity, as indicated by observed species, Shannon, Chao1, and ACE indices (**Supplementary Figures S2A–D**). Moreover, no significant differences were observed in overall microbial structure among groups (**Supplementary Figure S2E**). However, ISO treatment significantly modulated the gut microbial composition altered by MPTP at both phylum and genus levels (**Figures 2A, B**). Specifically, MPTP increased the relative abundance of Pseudomonadota and decreased that of Bacillota, whereas ISO treatment reversed these shifts (**Figure 2A**). At the genus level, the ISO-free + MPTP group exhibited increased abundances of *Ligilactobacillus*, *Escherichia-Shigella*, *Ruminococcus*, Prevotellaceae_NK3B31_group, and *Klebsiella*, along with reduced *Lactobacillus* abundance compared to the ISO-free group; these alterations were reversed by ISO supplementation (**Figure 2B**). In recipient mice, alpha diversity and overall microbial structure also showed no significant differences across groups (**Supplementary Figures S2F–J**). Consistent with donor mice, ISOF attenuated the MPTP-induced increase in Pseudomonadota and decrease in Bacillota (**Figure 2C**). The MPTP-induced increases in *Ligilactobacillus*, *Escherichia-Shigella*, and *Klebsiella*, as well as the reduction in *Lactobacillus*, were similarly reversed by ISOF (**Figure 2D**). LEfSe analysis further confirmed that MPTP reduced *Lactobacillus* abundance, an effect reversed by both ISO and ISOF treatments (**Figures 2E–G**). At the species level, *L. intestinalis* abundance was decreased by MPTP but restored by ISO and ISOF interventions (**Figures 2H, I**; **Supplementary Figure S2K**). These findings were validated by qPCR (**Supplementary Figures S3A, B**). To determine whether the protective effect of ISO is associated with *L. intestinalis*, we supplemented mice with *L. intestinalis* (**Figure 2J**). We first confirmed that *L. intestinalis* administration significantly increased fecal abundance of this species (**Figure 2K**). Behavioral tests revealed that *L. intestinalis* treatment markedly alleviated MPTP-induced motor deficits (**Supplementary Figures S3C, D**). Furthermore, *L. intestinalis* intervention significantly attenuated the loss of TH^+^ neurons in both the STR and SN (**Figures 2L–N**). Collectively, these results indicate that ISO-mediated enrichment of *L. intestinalis* contributes to the alleviation of the MPTP-induced PD-model in mice.

### 3.3 *L. intestinalis* alleviates the MPTP-induced PD-model by promoting 5-HT production

**Figure 3:**
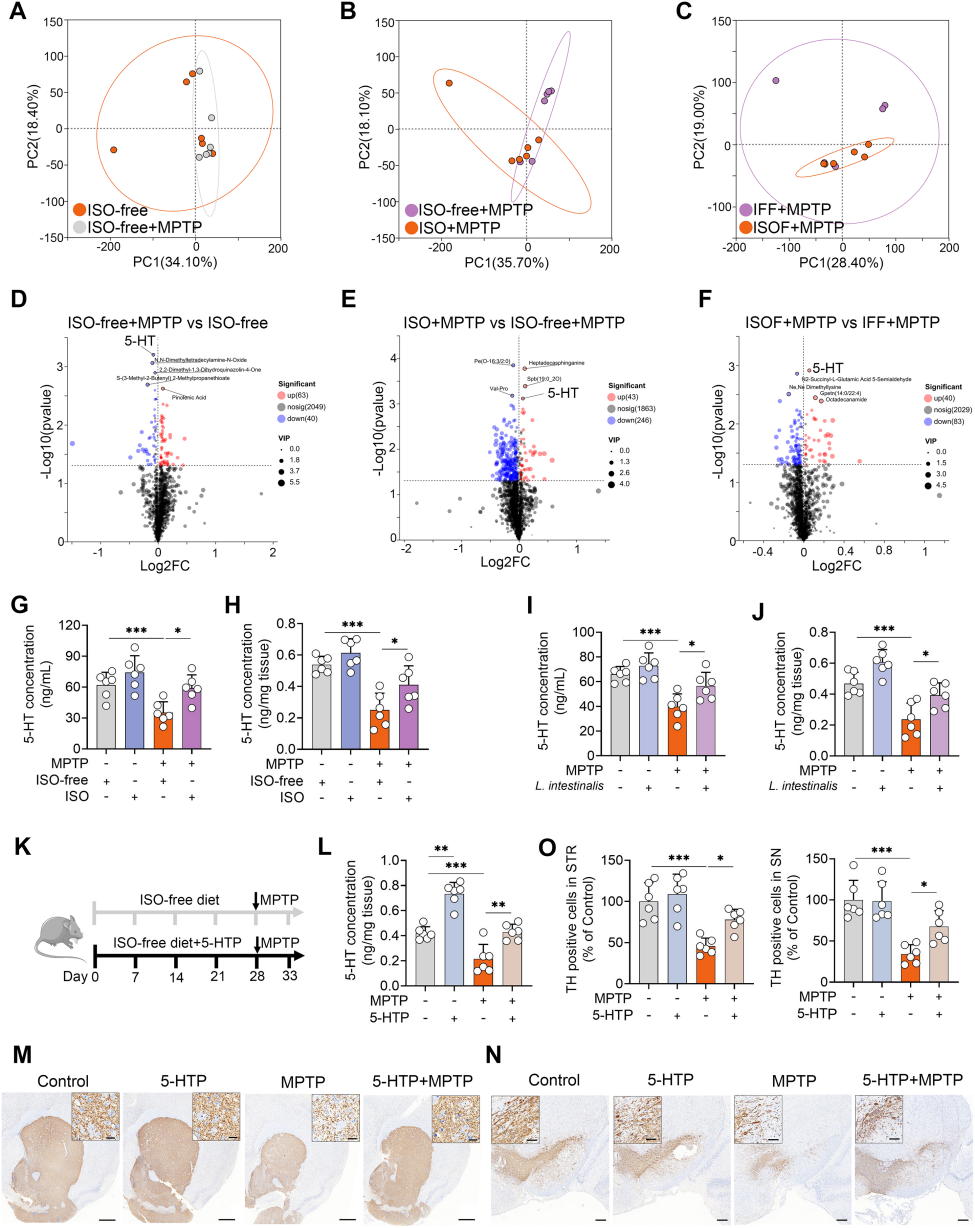
*L. intestinalis* alleviates MPTP-induced PD through increased 5-HT levels. **(A–C)** PCA score plots of serum metabolites across different comparison groups (n = 6). **(D–F)** Volcano plots showing differentially regulated metabolites between comparison groups: ISO-free + MPTP *vs*. ISO-free (**D**; 63 upregulated, 40 downregulated), ISO + MPTP *vs*. ISO-free + MPTP (**E**; 43 upregulated, 246 downregulated), and ISOF + MPTP *vs*. IFF + MPTP (**F**; 40 upregulated, 83 downregulated). **(G, H)** Serum **(G)** and striatal **(H)** 5-HT levels in mice from ISO treatment groups (n = 6). **(I, J)** Serum **(I)** and striatal **(J)** 5-HT levels in mice from *L. intestinalis* treatment groups (n = 6). **(K)** Schematic diagram of 5-HTP treatment. Mice were administered 5-HTP at a dose of 1 g/kg/day via food pellets for four weeks (n = 6). **(L)** Striatal 5-HT levels in 5-HTP-treated mice (n = 6). **(M, N)** Representative TH-stained images in the STR **(M)** and SN **(N)** from 5-HTP-treated mice. Scale bars: 500 μm for STR (inset, 20 μm) and 200 μm for SN (inset, 50 μm). **(O)** Quantification of TH-positive cells in the STR and SN. Data are expressed as mean ± SD (n = 6). *p < 0.05, **p < 0.01, and ***p < 0.001 by one-way ANOVA followed by Tukey’s *post hoc* test **(G–J, L, O)**.

Gut microbial metabolites play a crucial role in mediating the interaction between the gut microbiota and the host. We therefore investigated metabolic alterations induced by ISO supplementation. Principal component analysis (PCA) revealed that MPTP treatment significantly altered serum metabolic profiles compared to the ISO-free group (**Figure 3A**). Furthermore, both ISO and ISOF treatments shifted the serum metabolic composition relative to the ISO-free and IFF groups, respectively, under MPTP challenge (**Figures 3B, C**), a finding corroborated by PLS-DA score plots (**Supplementary Figures S3E–G**). Metabolomic profiling showed that mice fed an ISO-free diet exhibited 103 differential metabolites (63 upregulated, 40 downregulated), among which 5-HT had the lowest p-value (**Figure 3D**). Compared to the ISO-free group, ISO treatment led to 43 upregulated and 216 downregulated metabolites following MPTP administration (**Figure 3E**). Similarly, the ISOF group displayed 40 upregulated and 83 downregulated metabolites relative to the IFF group under MPTP treatment (**Figure 3F**). Notably, the MPTP-induced reduction in serum 5-HT levels was reversed by both ISO and ISOF interventions (**Figures 3E, F**). Further validation showed that serum 5-HT levels were significantly lower in the ISO-free + MPTP group than in the ISO-free control group, and this deficit was restored by ISO supplementation (**Figure 3G**). Interestingly, a parallel decrease in 5-HT concentration was observed in the STR, and these changes in both serum and STR were recapitulated in recipient mice (**Supplementary Figure S3H**). Importantly, treatment with *L. intestinalis* also mitigated the decline in 5-HT levels in both serum and STR (**Figures 3I, J**). To evaluate the functional impact of elevated 5-HT on MPTP-induced PD, we supplemented mice with the 5-HT precursor 5-HTP (**Figure 3K**). 5-HTP treatment significantly increased 5-HT levels in the STR and counteracted the MPTP-induced 5-HT reduction (**Figure 3L**). Moreover, 5-HTP ameliorated MPTP-induced motor deficits (**Supplementary Figures S3I, J**) and attenuated the loss of TH^+^ neurons in both the STR and SN (**Figures 3M–O**). Collectively, these findings indicate that *L. intestinalis* alleviates the MPTP-induced PD-model likely through enhancing 5-HT production.

### 3.4 5-HT attenuates the MPTP-induced PD-model by inhibition of ferroptosis

**Figure 4:**
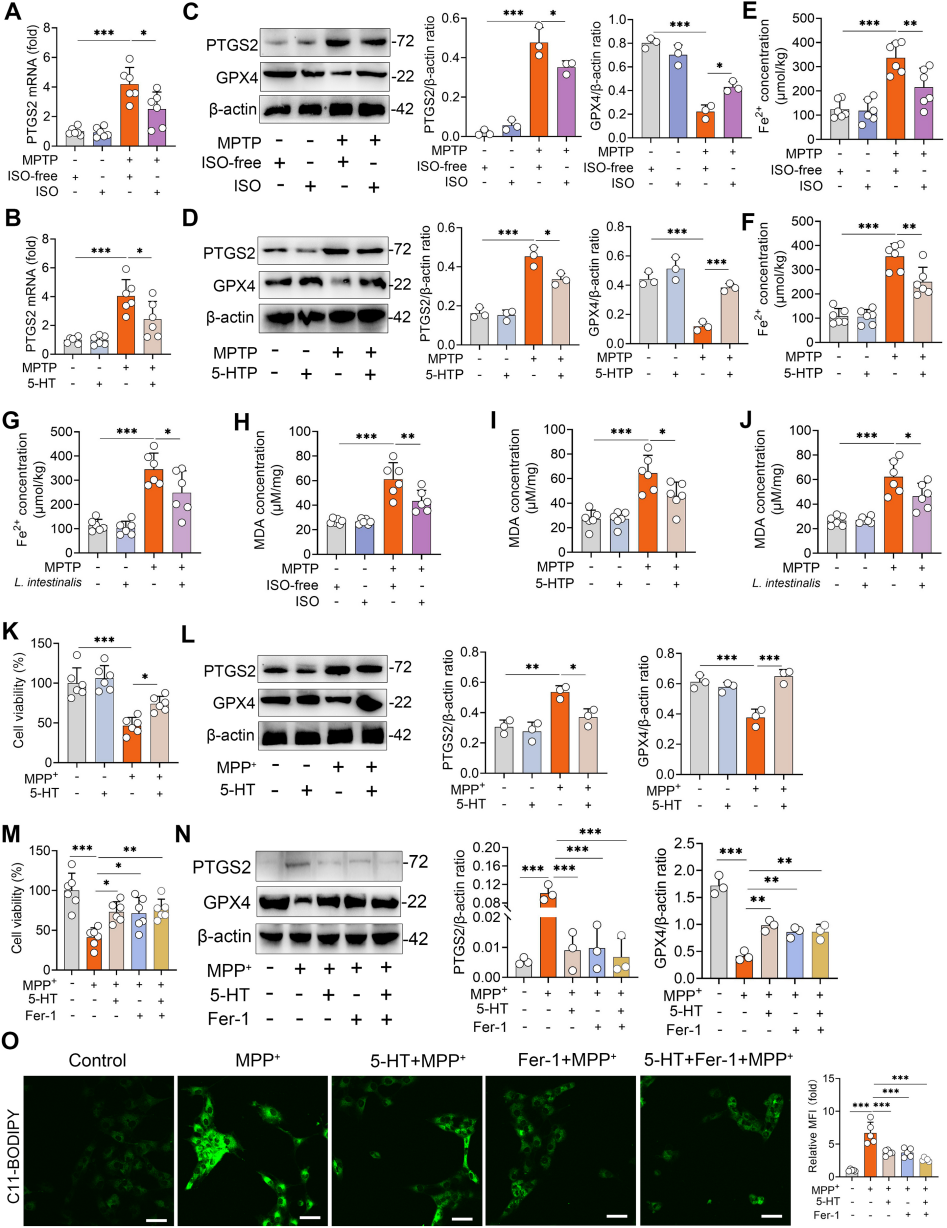
5-HT limits MPTP-induced ferroptosis. **(A, B)** PTGS2 mRNA levels in the STR from ISO-treated **(A)** and 5-HTP-treated mice **(B)**. **(C, D)** Representative Western blots of PTGS2 and GPX4 in STR tissues from ISO-treated **(C)** and 5-HTP-treated mice **(D)**, with quantitative intensity analysis (n = 3). **(E–G)** Fe²^+^ levels in the STR of ISO-treated **(E)**, 5-HTP-treated **(F)**, and *L. intestinalis*-treated mice **(G)**. **(H–J)** MDA levels in the STR of ISO-treated **(H)**, 5-HTP-treated **(I)**, and *L. intestinalis*-treated mice **(J)**. **(K, L)** Cells were pretreated with 10 μM 5-HT for 2 h prior to MPP^+^ exposure (1 mM). **(K)** Cell viability was assessed (n = 6). **(L)** Representative Western blots of PTGS2 and GPX4 and corresponding intensity analysis. **(M–O)** Cells were pretreated with 10 μM 5-HT and Fer-1 (10 μM) for 2 h before MPP^+^ (1 mM) treatment (n = 6). **(M)** Cell viability was measured (n = 6). **(N)** Representative Western blots of PTGS2 and GPX4 and intensity analysis in the indicated groups. **(O)** Lipid ROS levels were detected by C11-BODIPY 581/591 staining using confocal microscopy. Data are expressed as mean ± SD. *p < 0.05 **p < 0.01, and ***p < 0.001 by one-way ANOVA followed by Tukey’s *post hoc* test **(A–N)**.

Ferroptosis is a form of regulated cell death characterized by iron accumulation and excessive lipid peroxidation, and has been implicated in the pathogenesis of PD (25). We therefore investigated whether the protective effects of ISO-derived 5-HT are associated with the modulation of ferroptosis. First, we found that MPTP-treated mice exhibited increased PTGS2 mRNA levels compared to naïve controls, and these elevations were reversed by ISO treatment (**Figure 4A**). Similarly, both *L. intestinalis* administration and 5-HTP supplementation attenuated the MPTP-induced upregulation of PTGS2 mRNA (**Figure 4B**; **Supplementary Figure S4A**). Furthermore, MPTP treatment increased PTGS2 protein expression while reducing GPX4 levels in the STR, alterations that were counteracted by ISO, *L. intestinalis*, and 5-HTP interventions (**Figures 4B, C**; **Supplementary Figure S4B**). MPTP also elevated intracellular Fe²^+^ levels, which were normalized by ISO, *L. intestinalis*, and 5-HTP treatments (**Figures 4E–G**). In addition, MPTP increased malondialdehyde (MDA) levels—a key marker of oxidative lipid damage during ferroptosis—effects that were similarly reversed by all three treatments (**Figures 4H–J**). Moreover, MPTP reduced the expression of GSH, whereas ISO, *L. intestinalis*, and 5-HTP restored GSH levels (**Supplementary Figures S4C–E**). *In vitro*, MPP^+^ treatment significantly decreased cell viability compared to control cells, while 5-HT treatment ameliorated MPP^+^-induced cytotoxicity (**Figure 4K**). MPP^+^ also increased PTGS2 and decreased GPX4 protein levels relative to controls (**Figure 4L**), and 5-HT treatment reduced the MPP^+^-induced rise in MDA (**Supplementary Figure S4F**). To determine whether the neuroprotective effects of 5-HT depend on ferroptosis inhibition, we co-treated cells with 5-HT and the specific ferroptosis inhibitor ferrostatin-1 (Fer-1). Both 5-HT and Fer-1 individually alleviated MPP^+^-induced cell death, but no additive protective effect was observed in the co-treatment group (**Figure 4M**). Likewise, both agents improved MPP^+^-induced dysregulation of PTGS2 and GPX4, yet combination treatment showed no significant enhancement compared to either agent alone (**Figure 4N**). Similarly, both 5-HT and Fer-1 reduced MPP^+^-induced lipid peroxidation, but their combined application yielded effects comparable to monotherapy (**Figure 4O**). Collectively, these findings demonstrate that 5-HT attenuates the MPTP-induced PD-model by inhibiting ferroptosis.

### 3.5 5-HT limits ferroptosis by regulating the PI3K-AKT pathway

**Figure 5:**
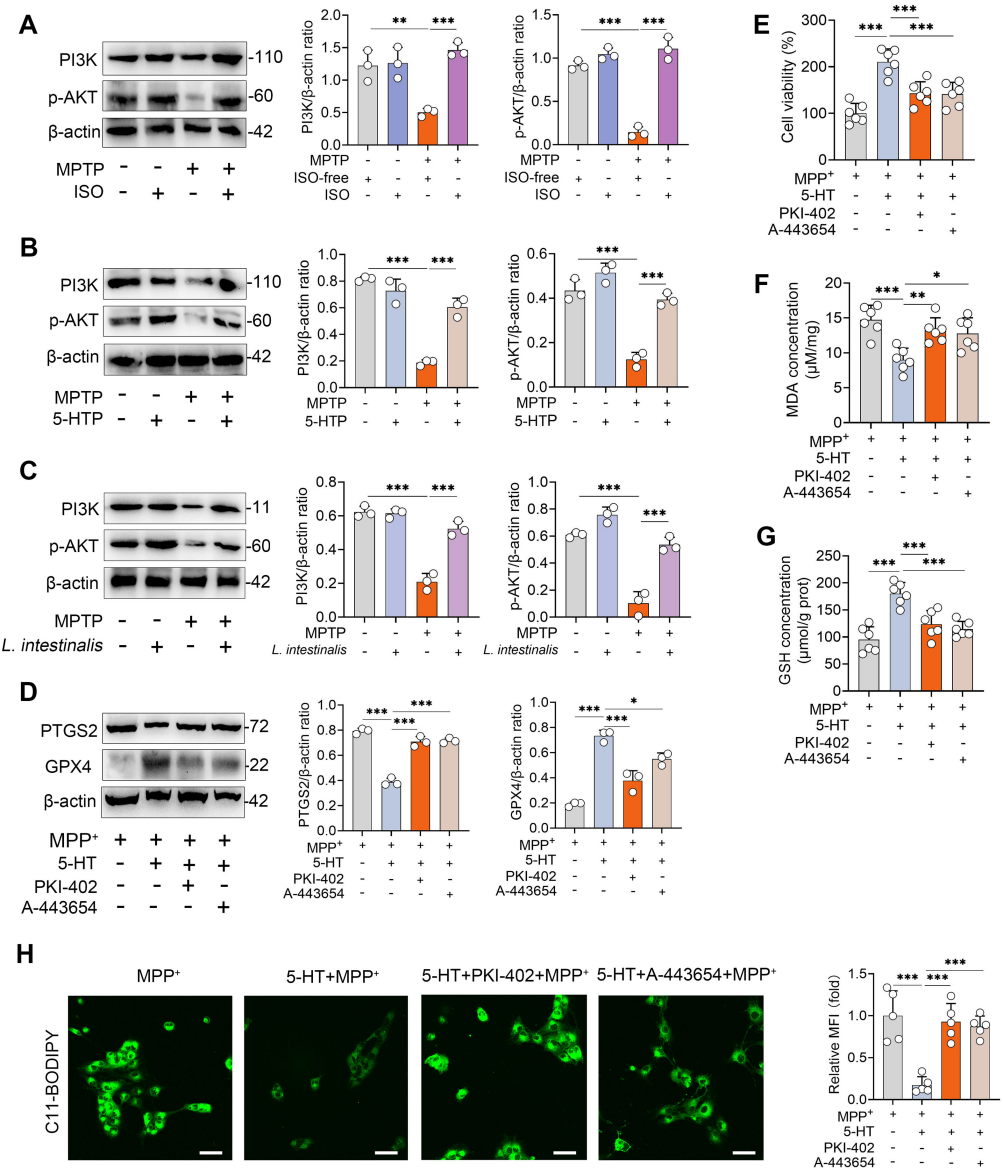
5-HT suppresses ferroptosis by regulating the PI3K-AKT signaling pathway. **(A–C)** Representative Western blots of PI3K and p-AKT in the STR from ISO-, 5-HTP-, and *L. intestinalis*-treated mice, with quantitative intensity analysis (n = 3). **(D–G)** Cells were pretreated with PKI-402 (1 μM) and A-443654 (10 μM) in combination with 10 μM 5-HT for 2 h prior to exposure to MPP^+^ (1 mM) (n = 6). **(D)** Representative Western blots of PTGS2 and GPX4 in the indicated groups and corresponding intensity analysis (n = 3). **(E)** Cell viability was assessed (n = 6). **(F, G)** MDA and GSH levels were measured (n = 6). **(H)** Lipid ROS levels were detected using C11-BODIPY 581/591 staining and confocal microscopy. Data are expressed as mean ± SD (n = 3–6). *p < 0.05, **p < 0.01, and ***p < 0.001 by one-way ANOVA followed by Tukey’s *post hoc* test **(A–G)**.

The PI3K-AKT signaling pathway has been identified as a key regulator of both ferroptosis and the pathogenesis of PD (28). We therefore investigated whether this pathway mediates the protective effects of 5-HT against ferroptosis. MPTP treatment significantly reduced the expression levels of PI3K and phosphorylated AKT (p-AKT), whereas ISO intervention restored these downregulated proteins (**Figure 5A**). Similarly, supplementation with 5-HTP attenuated the MPTP-induced reduction in PI3K and p-AKT levels (**Figure 5B**). Comparable results were observed in mice treated with *L. intestinalis* (**Figure 5C**). To confirm the involvement of the PI3K-AKT pathway in 5-HT-mediated suppression of ferroptosis, we used the specific inhibitors PKI-402 (for PI3K) and A-443654 (for AKT) (28, 38). We first confirmed that PKI-402 treatment attenuated the 5-HT-induced increases in PI3K and p-AKT under MPP^+^ treatment conditions (**Supplementary Figure S5A**). Furthermore, A-443654 reversed the 5-HT-mediated elevation of p-AKT in MPP^+^-treated cells (**Supplementary Figure S5A**). Notably, treatment with PKI-402 or A-443654 diminished the 5-HT-induced increase in GPX4 and decrease in PTGS2 under MPP^+^ challenge (**Figure 5D**). Consistently, inhibition of the PI3K-AKT pathway abolished the protective effect of 5-HT against MPP^+^-induced cell death (**Figure 5E**). Furthermore, the beneficial effects of 5-HT on antioxidant defenses—specifically, the reduction of MDA and elevation of GSH—were reversed by PKI-402 and A-443654 (**Figures 5F, G**). Additionally, blockade of the PI3K-AKT pathway attenuated the ability of 5-HT to suppress MPP^+^-induced lipid peroxidation (**Figure 5H**). These findings collectively indicate that 5-HT exerts its anti-ferroptotic effects through activation of the PI3K-AKT signaling pathway.

### 3.6 5-HT inhibits ferroptosis by activating the 5-HTR1A-PI3K-AKT pathway

**Figure 6:**
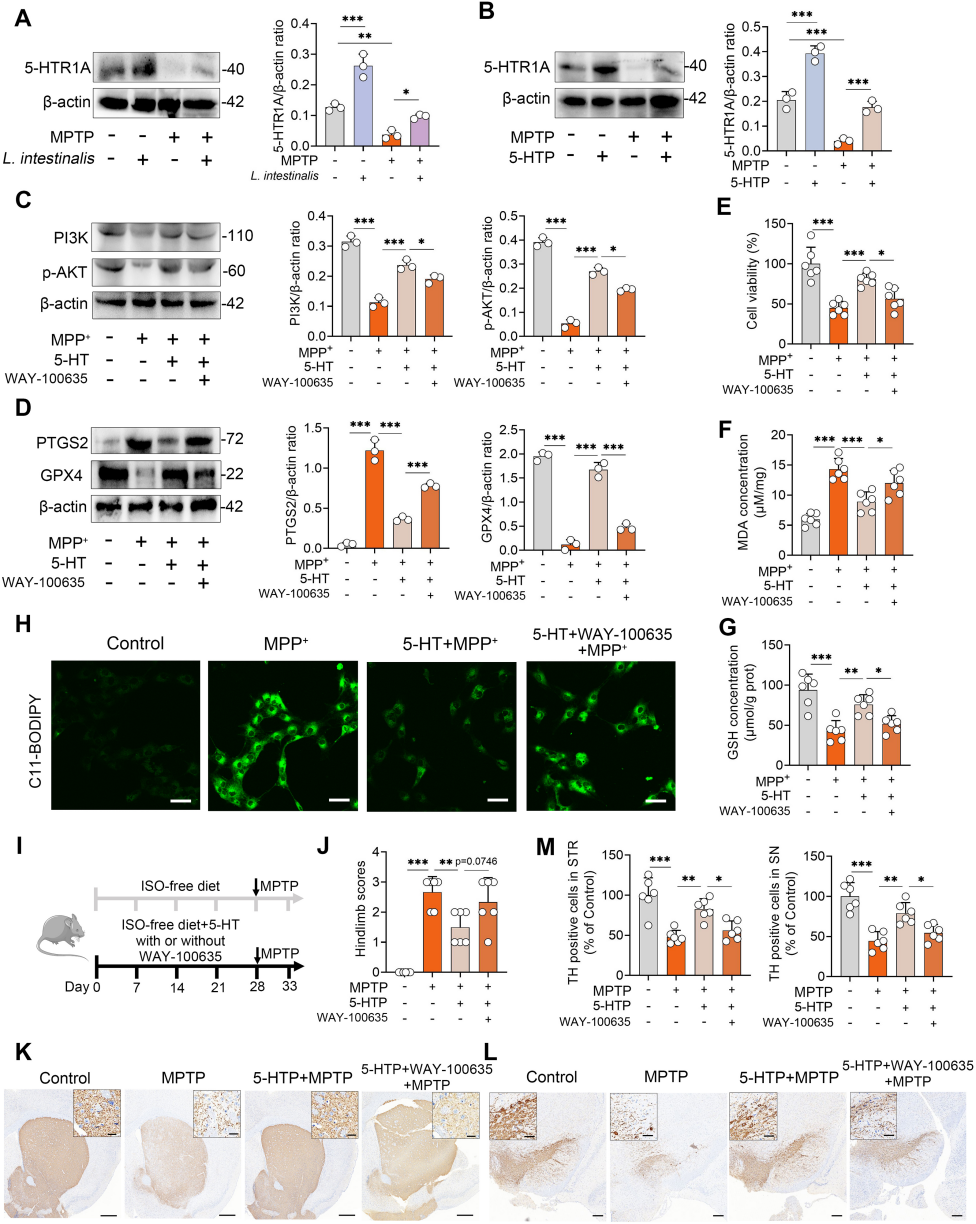
5-HT inhibits ferroptosis by activating the 5-HTR1A-PI3K-AKT signaling pathway. **(A, B)** Representative Western blots of 5-HTR1A in the STR from *L. intestinalis*- and 5-HTP-treated mice, with quantitative intensity analysis. **(C–H)** Cells were pretreated with WAY-100635 (1 μM) in combination with 10 μM 5-HT for 2 h prior to MPP^+^ (1 mM) exposure (n = 3). **(C)** Representative Western blots of PI3K and p-AKT in the indicated groups and corresponding intensity analysis (n = 3). **(D)** Representative Western blots of PTGS2 and GPX4 and intensity analysis (n = 3). **(E–G)** Cell viability **(E)**, MDA levels **(F)**, and GSH levels **(G)**. **(H)** Lipid ROS levels were assessed using C11-BODIPY 581/591 staining and confocal microscopy. **(I)** Schematic diagram of 5-HTR1A inhibition. Mice received daily subcutaneous injection of WAY-100635 (1 mg/kg) alongside 5-HTP treatment for four weeks, followed by intraperitoneal injection of MPTP (15 mg/kg) for five consecutive days to induce PD (n = 6). **(J)** Hindlimb clasping scores. **(K, L)** Representative TH-stained images in the STR **(K)** and SN **(L)**. Scale bars: 500 μm for STR (inset, 20 μm) and 200 μm for SN (inset, 50 μm). **(M)** Quantification of TH-positive cells in the STR and SN. Data are expressed as mean ± SD (n = 6). *p < 0.05, **p < 0.01, and ***p < 0.001 by one-way ANOVA followed by Tukey’s *post hoc* test **(A–G, J, M)**.

We next investigated through which mechanism 5-HT regulates the PI3K-AKT pathway-mediated ferroptosis. The 5-HTR1A is highly expressed in striatal neurons and can activate the downstream PI3K-AKT signaling pathway (36), which has been negatively correlated with the progression of PD (29). Therefore, we further examined whether the anti-ferroptotic effect of 5-HT is mediated by activation of the 5-HT1A receptor. The results showed that MPTP treatment reduced the expression of the 5-HT1A receptor, whereas both *L. intestinalis* and 5-HTP treatments increased 5-HT1A levels and reversed the MPTP-induced downregulation of this receptor (**Figures 6A, B**). To test this mechanistic link, we inhibited 5-HTR1A using the specific antagonist WAY100635 (36, 45), and found that the 5-HT-induced increases in PI3K and p-AKT were abolished by WAY100635 *in vitro* (**Figure 6C**). Moreover, inhibition of 5-HTR1A attenuated the protective effects of 5-HT against ferroptosis, as evidenced by elevated PTGS2 and reduced GPX4 levels following WAY100635 treatment compared to 5-HT alone (**Figure 6D**). Similarly, WAY100635 completely abolished the improvement in cell viability conferred by 5-HT under MPP^+^ challenge (**Figure 6E**). Additionally, the beneficial effects of 5-HT on antioxidant function—including suppression of MDA accumulation and enhancement of GSH levels—and its inhibition of lipid peroxidation were diminished upon 5-HT1A receptor blockade (**Figures 6F-H**; **Supplementary Figure S5B**). We further validated the role of the 5-HT1A receptor *in vivo* and found that pharmacological inhibition of 5-HT1A attenuated the protective effects of 5-HTP on MPTP-induced motor dysfunction (**Figures 6I, J**; **Supplementary Figures S5C–F**). Consistently, the increases in TH^+^ neuron expression in both the STR and SN induced by 5-HTP were reversed by WAY100635 treatment (**Figures 6K–M**). Collectively, these findings demonstrate that 5-HT inhibits ferroptosis via activation of the 5-HTR1A-PI3K-AKT signaling pathway.

## 4 Discussion

PD is a prevalent neurodegenerative disorder, and to date, no effective strategies for its prevention or treatment have been established. Different dietary patterns, particularly dietary isoflavones, have been reported to be negatively associated with the risk of PD. Additionally, accumulating evidence indicates that gut dysbiosis plays a critical role in the pathogenesis of PD (6, 7), and probiotic supplementation has shown potential benefits for PD prevention (9). However, it remains unclear whether dietary isoflavones can alleviate PD through modulation of the gut microbiota. In this study, we demonstrate that an isoflavone-enriched diet alleviates MPTP-induced PD in mice in a gut microbiota-dependent manner. Specifically, isoflavones promote the expansion of intestinal *Lactobacillus*, which enhances 5-HT production in the brain. Mechanistically, 5-HT inhibits MPTP-induced ferroptosis by activating the 5-HTR1A receptor and subsequently the PI3K-AKT signaling pathway.

Diet is one of the most important factors shaping the gut microbiota. Among dietary components, isoflavones can modulate gut microbial composition and exert significant neuroprotective effects (10, 12). For example, in a rotenone-induced mouse model of PD, treatment with isoflavone-rich extracts or individual isoflavones rescued the loss of dopaminergic neurons and reversed neurite shortening in primary mesencephalic cultures (46). Puerarin has demonstrated therapeutic efficacy in both clinical and experimental studies through multiple mechanisms, including antioxidant activity, attenuation of inflammatory responses, and elevation of dopamine and its metabolite levels (47). Moreover, daidzein exerts neuroprotective effects against MPTP-induced PD by suppressing microglial inflammation (48). Consistent with these findings, we found that a daidzein- and genistein-enriched isoflavone diet also alleviated MPTP-induced PD. Notably, our study reveals that the protective effects of isoflavones against MPTP-induced PD are at least partially dependent on the gut microbiota, as evidenced by antibiotic depletion experiments and FMT assays. Zhao et al. similarly reported that FMT from healthy donors alleviated rotenone-induced PD in mice by suppressing lipopolysaccharide-TLR4-mediated inflammation via the microbiota-gut-brain axis (49). Furthermore, we observed increased abundance of *Lactobacillus* and reduced levels of opportunistic pathogens such as *Escherichia-Shigella* and *Klebsiella* in mice receiving ISO treatment. A decrease in fecal *Lactobacillus* abundance has also been observed in rotenone-induced PD and is closely associated with gastrointestinal dysfunction and motor symptoms (50). Administration of *L. intestinalis* alleviated MPTP-induced deficits in mice, which aligns with previous evidence indicating that *Lactobacillus* species are potential probiotics for PD intervention (32, 51). For instance, *Lactobacillus plantarum* DP189 attenuated PD symptoms in mice by modulating oxidative damage, inflammation, and gut microbiota dysbiosis (51). Similarly, *Lactobacillus reuteri* ameliorated MPTP-induced PD by inhibiting ferroptosis through the AKT-GSK3β-GPX4 pathway, mediated by GABA production (32).

Metabolic alterations represent a common mechanism underlying the interaction between host and gut microbiota. We next found that isoflavone supplementation reversed MPTP-induced metabolic disturbances in the serum, particularly by restoring 5-HT levels. Notably, this effect was mediated by the gut microbiota, as both ISOF and *L. intestinalis* treatment also increased 5-HT levels. Studies have reported that, in addition to their direct effects, many isoflavones can be further metabolized by the gut microbiota into secondary metabolites, which are more efficiently absorbed by the intestine and capable of exerting biological effects in distant organs. For instance, the gut microbial metabolite of quercetin, 3,4-dihydroxyphenylacetic acid (DOPAC), enhances CD8^+^ T cell-mediated anti-tumor immunity through Nrf2-mediated mitophagy (52). DOPAC liberated by *Streptococcus thermophilus* has also been shown to alleviate polycystic ovary syndrome via β-galactosidase activity (53). Moreover, berberine ameliorates ovariectomy-induced anxiety-like behaviors by promoting equol production through modulation of the gut microbiota (54). Chu et al. demonstrated that *Lactobacillus plantarum* CCFM405 elevated dopamine, 5-HT, and associated metabolites in the striatum of PD mice (55). *Lactobacillus acidophilus*-derived components reduce intestinal inflammation and increase 5-HT levels in the SN during PD (56). Other studies have similarly shown that flavonoid extracts alleviate rotenone-induced PD and enhance brain 5-HT levels (55, 57). However, most of these studies have merely documented changes in 5-HT without investigating its functional role in PD pathogenesis. We further demonstrated that enhancing 5-HT levels through 5-HTP supplementation alleviates MPTP-induced PD, which aligns with previous findings showing that serotonin mitigates depression in a rotenone-induced mouse model of PD by inhibiting hippocampal neuronal pyroptosis and neuroinflammation (58). Other studies have also demonstrated that gut microbiota-induced elevation of 5-HT serves as a key regulator in mediating the gut–brain interaction during autism spectrum disorders and Alzheimer’s disease (59, 60).

Ferroptosis has been closely associated with the development of PD (25, 27). Dong et al. recently demonstrated that formononetin derived from *Parabacteroides merdae* alleviates ferroptosis in PD mice by modulating the PI3K-AKT signaling pathway (61). Similarly, we found that isoflavones, *L. intestinalis*, and 5-HT alleviated MPTP-induced ferroptosis both *in vivo* and *in vitro*. Studies have also reported that *Lactobacillus* species mitigate ferroptosis through multiple mechanisms (16, 32, 62). For example, GABA derived from *Lactobacillus reuteri* alleviates MPTP-induced ferroptosis via the AKT-GSK3β-GPX4 axis in PD mice (32). Daidzein derived from *Lactobacillus* has also been shown to attenuate ferroptosis by modulating the AKT-GSK3β-Nrf2 pathway during acute liver injury (16). Tryptophan metabolism-associated metabolites, particularly 5-HT, have recently been identified as anti-ferroptotic agents in tumor cells (30). In lung adenocarcinoma, 5-HT inhibits ferroptosis by activating the Ca²^+^-CAMKK2-AMPK pathway via the 5-HT3 receptor (63). Additionally, 5-HT can activate the 5-HT2 receptor, which cooperates with Fyn to directly regulate p85 activity and trigger the PI3K-Akt-mTOR signaling pathway, leading to increased expression of HIF1α and ABCD1, along with reduced lipid peroxidation and suppression of ferroptosis (64). In this study, we demonstrate that 5-HT activates the 5-HTR1A receptor, and that inhibition of this receptor attenuates the protective effects of 5-HT against ferroptosis and PD. Notably, activation of 5-HTR1A has been reported to improve core parkinsonian symptoms as well as side effects induced by antiparkinsonian agents (65). Furthermore, 5-HTR1A upregulates the PI3K-AKT pathway, which is consistent with previous findings showing that 5-HTR1A-mediated activation of the PI3K-AKT pathway exerts neuroprotective effects (36).

## 5 Conclusion

Collectively, our results demonstrate that ISO facilitates *L. intestinalis* expansion and enhances 5-HT production, leading to the inhibition of ferroptosis in dopaminergic neurons through activation of the PI3K-AKT pathway via 5-HTR1A, thereby alleviating MPTP-induced PD in mice. Our findings not only confirm the role of the gut microbiota in PD pathogenesis through the gut-brain axis, but also highlight the potential of dietary interventions targeting the gut microbiota for the prevention or treatment of PD.

### 5.1 Limitations of the study

Although 5-HTR1A was downregulated in MPTP-treated mice, both ISO-diet and 5-HTP restored its expression and improved PD-like symptoms. However, treatment with WAY-100635 attenuated the protective effects of ISO-diet against PD in mice, confirming the involvement of 5-HTR1A in ISO-diet- and 5-HTP-mediated protection. Nevertheless, we cannot exclude the possibility that other mechanisms may also contribute to ISO-diet-mediated neuroprotection, such as alternative cell death pathways, suppression of inflammatory responses, and modulation of 5-HT on synaptic function. Furthermore, validation in human cohorts is required to confirm the relationship between isoflavone intake, 5-HT production, and inhibition of ferroptosis in PD patients.

## Data Availability

The raw data supporting the conclusions of this article will be made available by the authors, without undue reservation.
