# Supplementary material for: An isoflavone-enriched diet alleviates Parkinson’s disease in mice by inhibiting ferroptosis through gut microbiota-mediated serotonin production
Source: Front Immunol. 2026 Feb 3;17:1730833. doi: 10.3389/fimmu.2026.1730833 (PMC12909490; doi:10.3389/fimmu.2026.1730833)
Supplement: Supplementary file 1 [file Table1.docx]

**Supplementary information for**

**An isoflavone-enriched diet alleviates Parkinson's disease by inhibiting ferroptosis through gut microbiota-mediated serotonin production**

Le Yang^1, 2*^, Yan Ma^2^, Haiying Wang^2^, Likai Xie^2^, Yan Yu^1*^

1. School of Public Health, Xi'an Jiaotong University Health Science Center, Xi'an Shaanxi, 710061, China.

2. Jilin Provincial People's Hospital‌，Changchun, 130021, Jilin Province, China.

^*^Corresponding author:

Le Yang: yangle800511@sina.com

Yan Yu: yuyan@mail.xjtu.edu.cn


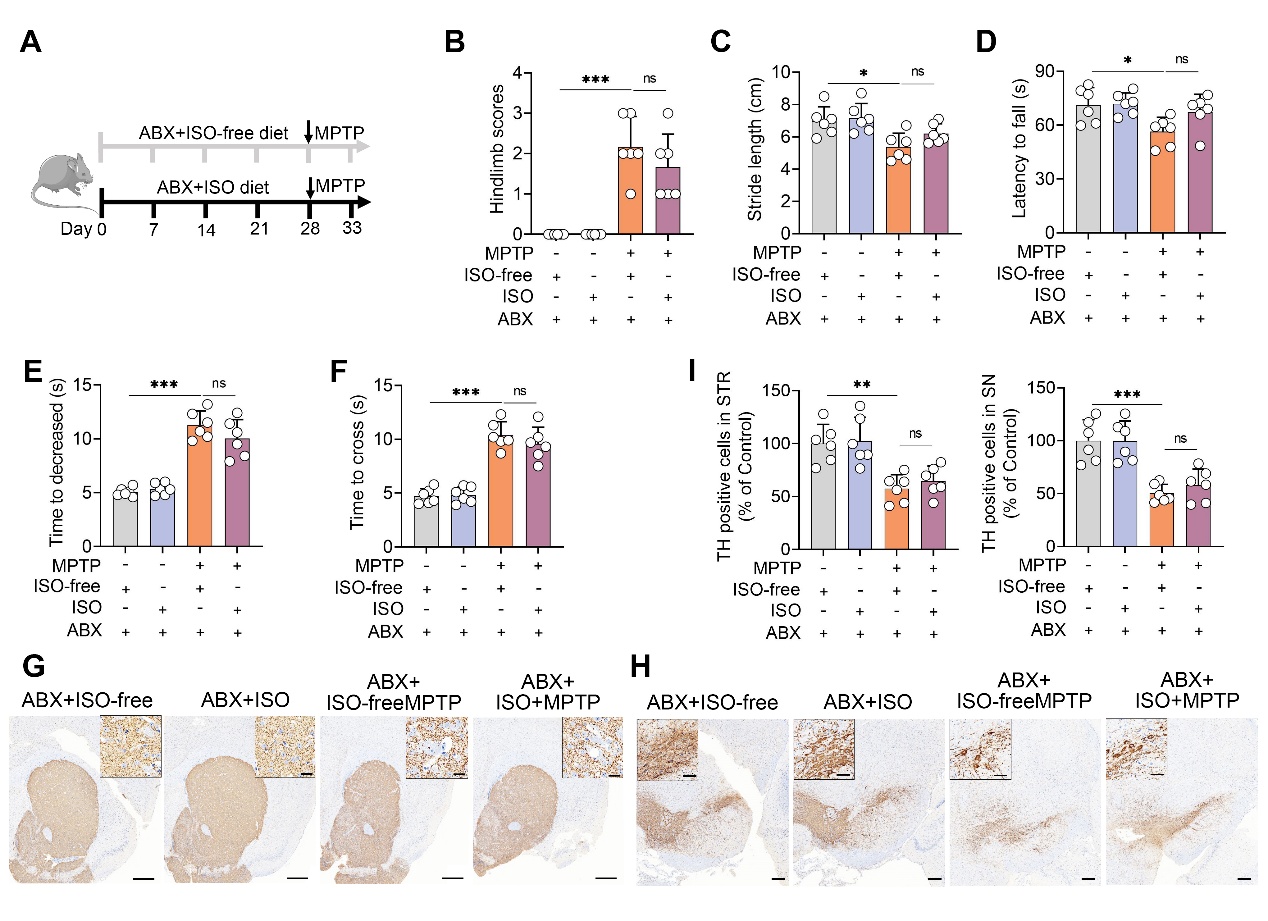


**Fig. S1. Antibiotic treatment abolishes the protective effect of the isoflavone diet against MPTP-induced PD in mice.** (A) Schematic diagram of isoflavone (ISO) diet intervention. Mice were administered sterile water supplemented with vancomycin (0.5 g/L), neomycin (1 g/L), metronidazole (1 g/L), and ampicillin (1 g/L) via drinking water for four weeks, concurrently with either an ISO diet or an ISO-free diet. This was followed by intraperitoneal injection of MPTP (15 mg/kg) for five consecutive days to induce PD (n = 6). (B–F) Behavioral assessments including hindlimb clasping scores (B), stride length (C), latency to fall (D), time to descend (E), and time to cross the beam (F). (G and H) Representative TH-stained images in the striatum (STR, G) and substantia nigra (SN, H). Scale bars: 500 μm for STR (inset, 20 μm) and 200 μm for SN (inset, 50 μm). (I) Quantification of TH-positive cells in the STR and SN. Data are expressed as mean ± SD (n = 6). **p* < 0.05, ***p* < 0.01, and ****p* < 0.001 by one-way ANOVA followed by Tukey’s post hoc test (B–F, I).


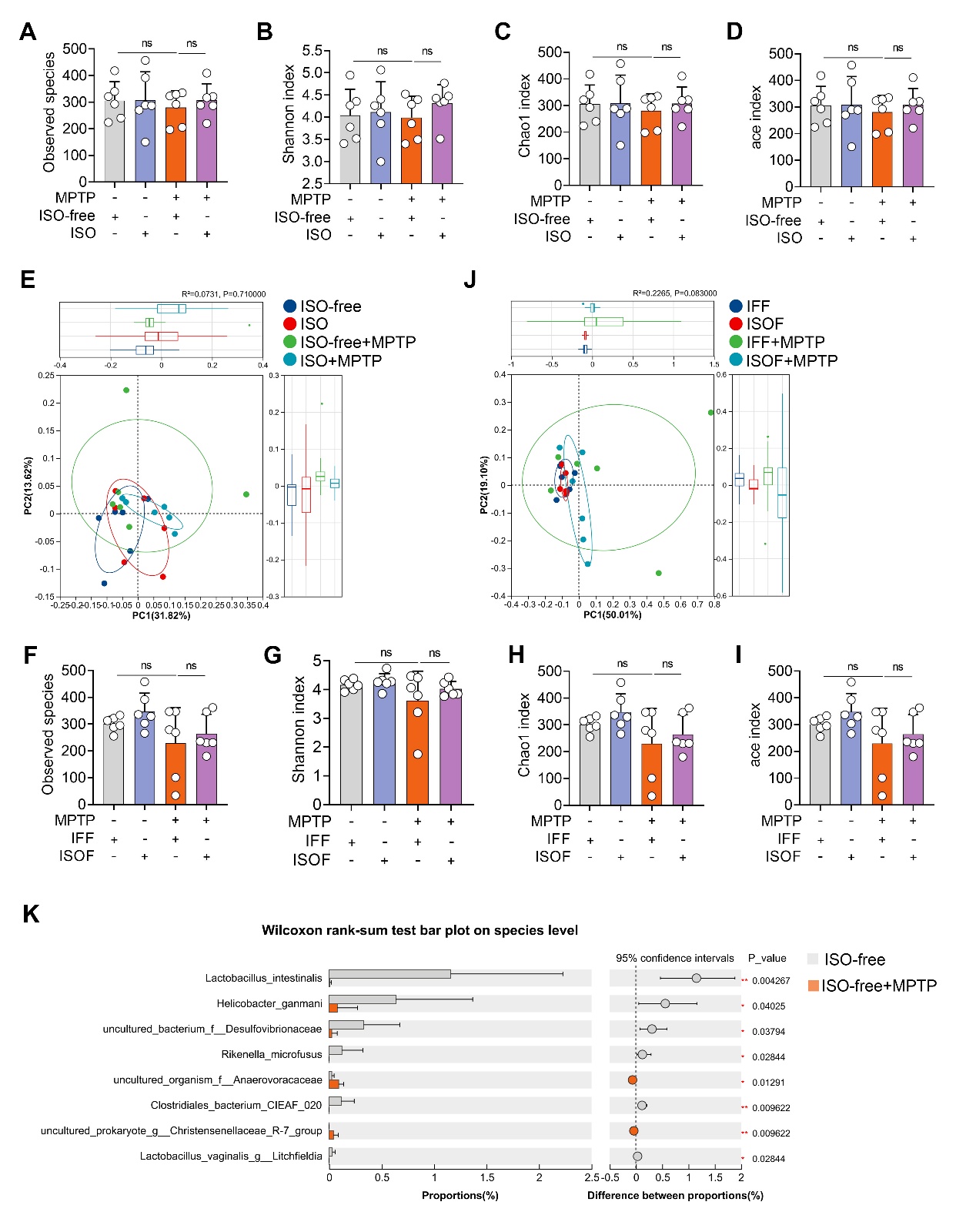


**Fig. S2. Isoflavone diet alters gut microbiota composition in mice.** (A–D) Alpha diversity indices of gut microbiota in ISO-treated mice, including observed species (A), Shannon index (B), Chao1 (C), and ACE index (D). (E) PCoA score plot showing gut microbial community structure in ISO-treated mice. (F–I) Alpha diversity indices in FMT mice, including observed species (F), Shannon index (G), Chao1 (H), and ACE index (I). (J) PCoA score plot depicting microbial structural differences in FMT mice. (K) Student’s t-test identified differentially abundant bacterial species between experimental groups. Data are expressed as mean ± SD (n = 6). Statistical significance was determined by one-way ANOVA (A–D, F–I).


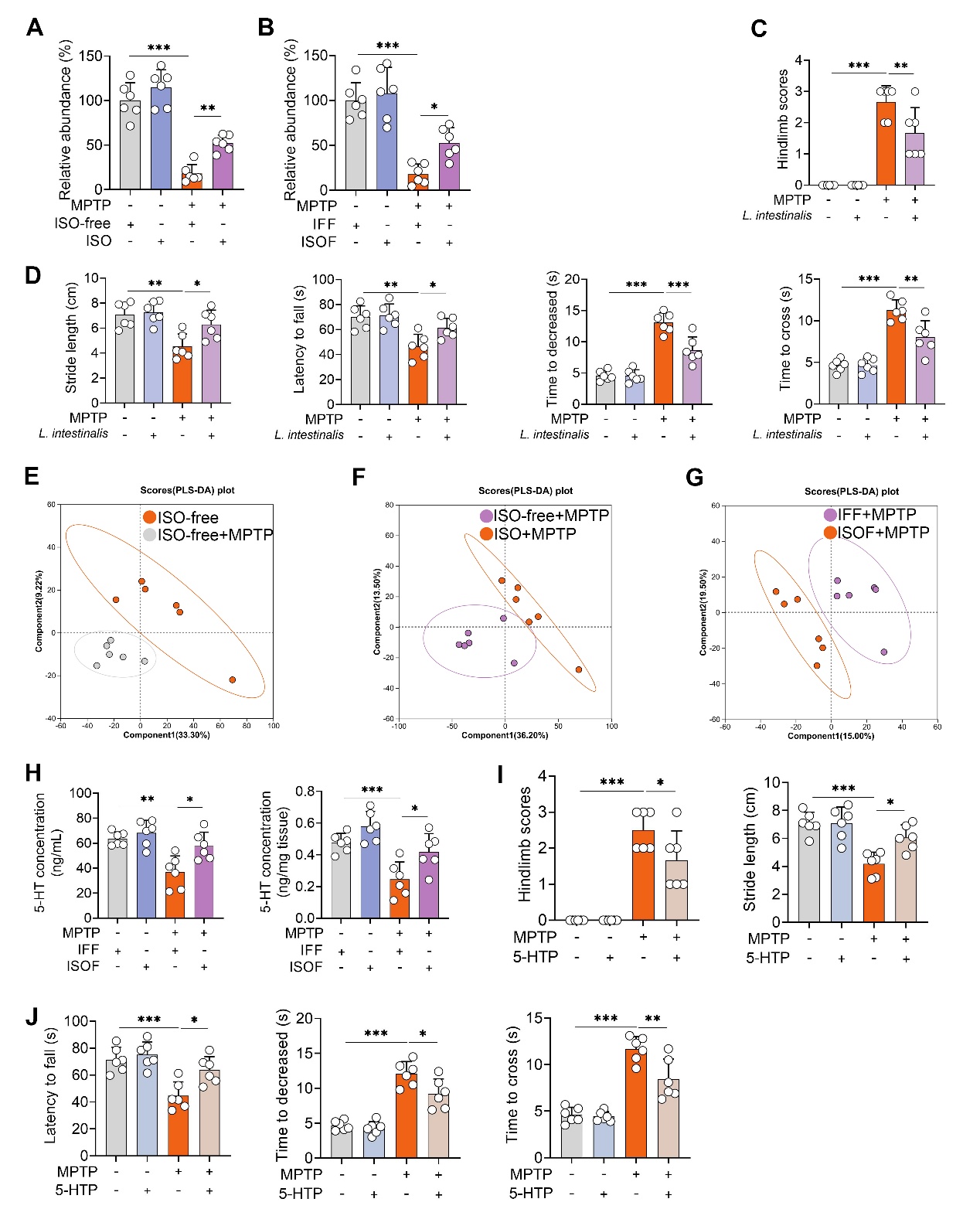


**Fig. S3. *L. intestinalis* and 5-HTP treatments alleviate MPTP-induced PD in mice.** (A and B) Fecal abundance of *L. intestinalis* in different mouse groups (n = 6). (C and D) Mice were administered antibiotics (200 mg/kg ampicillin, neomycin, and metronidazole; 100 mg/kg vancomycin) orally for five consecutive days to deplete commensal gut microbiota, followed by pretreatment with L. intestinalis (2 × 10⁸ CFU/mouse) for four weeks prior to MPTP administration (n = 6). Behavioral indices were assessed, including hindlimb clasping scores, stride length, latency to fall, time to descend, and time to cross the beam. (E–G) PLS-DA score plots of serum metabolites from the indicated mouse groups (n = 6). (H) Serum (left) and striatal (right) 5-HT levels were quantified (n = 6). (I and J) Mice received 5-HTP at a dose of 1 g/kg/day via food pellets for four weeks (n = 6), and behavioral indices were evaluated, including hindlimb clasping scores, stride length, latency to fall, time to descend, and time to cross the beam. Data are expressed as mean ± SD (n=6). **p* < 0.05, ***p* < 0.01 and ****p* < 0.001 by one-way ANOVA followed by Tukey’s test (A-D and H-J).


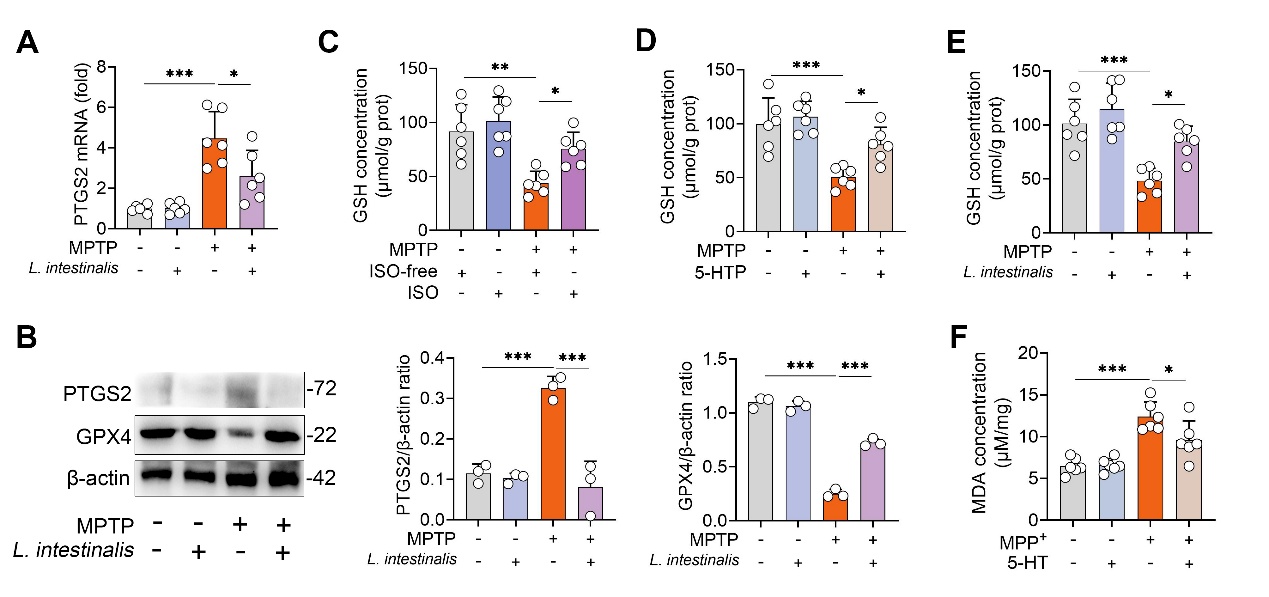


**Fig. S4. *L. intestinalis* limits MPTP-induced ferroptosis.** (A) PTGS2 mRNA levels in the STR of *L. intestinalis*-treated mice. (B) Representative Western blots of PTGS2 and GPX4 in STR tissues, with quantitative intensity analysis (n = 3). (C–E) GSH levels in the STR of ISO-treated (C), 5-HTP-treated (D), and *L. intestinalis*-treated mice (E). (F) Cells were pretreated with 10 μM 5-HT for 2 h prior to MPP⁺ exposure (1 mM), and MDA levels were measured (n = 6). Data are expressed as mean ± SD. **p* < 0.05, ***p* < 0.01 and ****p* < 0.001 by one-way ANOVA followed by Tukey’s post hoc test (A–F).


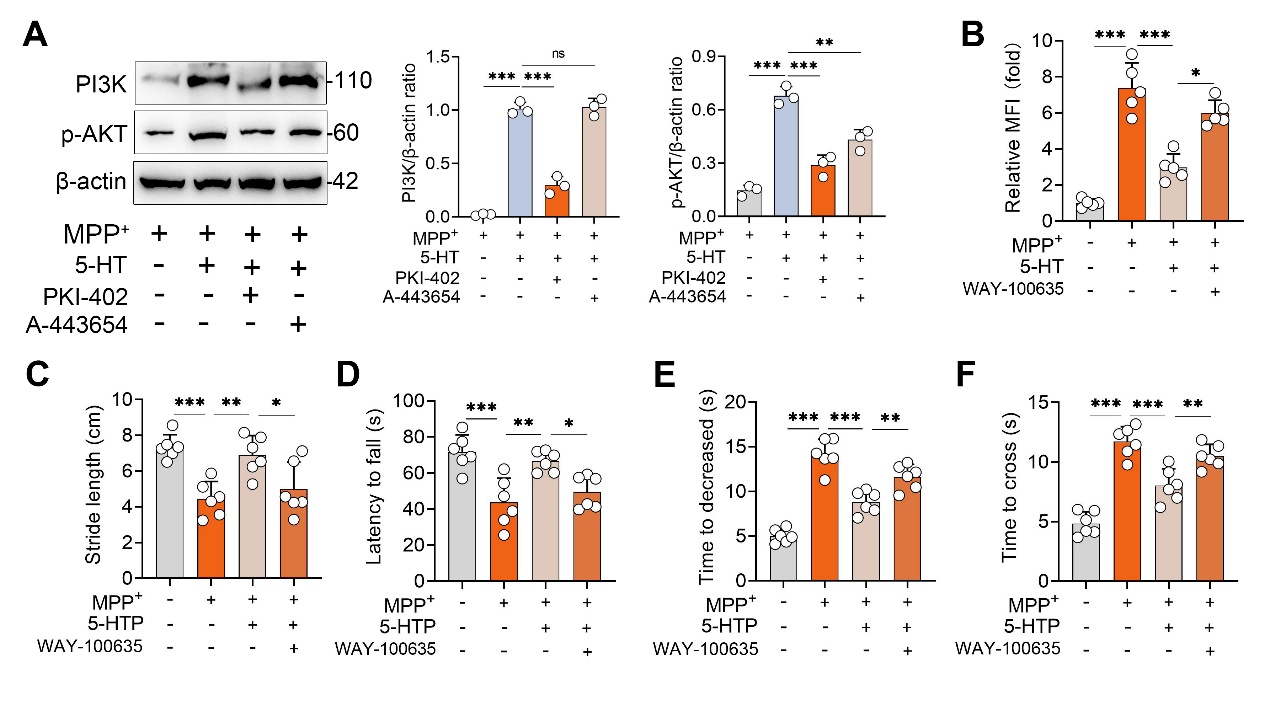


**Fig. S5. 5-HT alleviates PD by activating 5-HTR1A.** (A) Cells were pretreated with PKI-402 (1 μM) or A-443654 (10 μM) in combination with 5-HT (10 μM) for 2 h prior to MPP⁺ (1 mM) exposure, followed by western blot analysis to detect PI3K and p-AKT expression (n = 3). B. Relative MFI values derived from C11-BODIPY 581/591-stained images of the indicated cell treatments. C-F. Mice received daily subcutaneous injection of WAY-100635 (1 mg/kg) alongside 5-HTP treatment for four weeks, followed by intraperitoneal injection of MPTP (15 mg/kg) for five consecutive days to induce PD (n = 6). Behavioral indices were assessed, including stride length (C), latency to fall (D), time to descend (E), and time to cross the beam (F). Data are expressed as mean ± SD (n=6). **p* < 0.05, ***p* < 0.01 and ****p* < 0.001 by one-way ANOVA followed by Tukey’s test (A-F).
